# QTc interval prolongation in ICU patients: risk assessment and predictors

**DOI:** 10.1186/s43044-026-00713-y

**Published:** 2026-01-16

**Authors:** Muhammad Arsalan Sharif Awan, Bhavna Singla, Areeba Hasan, Mohamed Omer W. Abdalla, Rabia Altaf, Shivam Singla, Samia Afaq, Mian Waqar Mustafa, Fahad Asim

**Affiliations:** 1 Mayo Hospital, Lahore, Pakistan; 2 Internal Medicine, Erie County Medical Center, Buffalo, NY USA; 3 Liaqat national hospital and medical college, Karachi, Pakistan; 4 MBBS, Internal Medicine Resident, DALLAH HOSPITAL, Riyadh, Pakistan; 5 School of Pharmacy, University of Management and Technology, Lahore, Pakistan; 6 Tidal Health Peninsula Regional, Salisbury, Maryland USA; 7 Lecturer Faculty of Pharmacy, The University of Lahore, Lahore, Pakistan; 8 Assistant Professor Department of Pharmacy, Forman Christian College University, Lahore, Pakistan; 9 Lecturer in Pharmacology & Therapeutics, The University of Lahore, Lahore, Pakistan

**Keywords:** QTc prolongation, ICU, Critically ill, Sepsis, Haloperidol, Electrolyte imbalance, Mortality, Acute kidney injury

## Abstract

**Background:**

Prolonged corrected QTcs are a frequent and potentially life-threatening finding In ICU patients, the presence of prolonged QTc is a prevalent and potentially life-threatening condition, as it increases the risk of TdP and sudden cardiac death. Risk factors include sepsis, electrolyte imbalances, and QTc-prolonging drugs. However, data from South Asian ICUs remain limited.

**Methods:**

We conducted a 12-month prospective observational study in two tertiary care ICUs in Lahore and Karachi, enrolling 812 adult patients without pre-existing QTc abnormalities. QTc was calculated using Bazett’s formula, with prolongation defined as QTc ≥ 450ms (men) or QTc ≥ 470 ms (women). Clinical, biochemical, and pharmacologic data were collected. Multivariate logistic regression identified independent predictors of ICU-acquired QTc prolongation.

**Results:**

QTc prolongation occurred in 260 (32.0%) patients. These individuals had higher rates of sepsis (55%), acute kidney injury (45%), and electrolyte disturbances (hypokalemia 35%, hypomagnesemia 30%). QTc-prolonging medications, particularly haloperidol (22%), were more common among cases. Septic shock, coronary artery disease, renal dysfunction, and haloperidol were significant independent predictors. Prolonged QTc was associated with longer ICU stays (median 10 vs. 5 days) and higher mortality (45% vs. 18%, *p* < 0.001).

**Conclusion:**

In this inaugural multicenter study conducted in Pakistan, it was observed that one-third of patients in the ICU experienced QTc prolongation, which was associated with sepsis, renal dysfunction, and the administration of specific medications. These findings underscore the importance of QTc monitoring, electrolyte management, and cautious drug selection to reduce arrhythmia-related complications.

## Introduction

Prolongation of the heart rate–corrected QTc (QTc) on ECG reflects delayed ventricular repolarization and is a well-recognized harbinger of malignant ventricular arrhythmias, particularly TdP (TdP) [1]. This disturbance in cardiac electrophysiology is of major clinical concern because it can precipitate polymorphic ventricular tachycardia, syncope, and sudden cardiac death. Critically ill patients are especially vulnerable to QTc prolongation due to the convergence of multiple risk factors [2].

In intensive care settings, patients frequently receive several QTc-prolonging drugs—such as antiarrhythmics, fluoroquinolone antibiotics, macrolides, and antipsychotics—either simultaneously or sequentially. These agents can exert additive or synergistic effects on cardiac ion channels [3]. Moreover, metabolic and electrolyte derangements, particularly hypokalemia, hypomagnesemia, and hypocalcemia, further compound the risk of impaired repolarization [3, 4]. Acute organ dysfunction, including renal and hepatic impairment, alters drug metabolism and clearance, thereby potentiating drug accumulation and enhancing proarrhythmic effects. Patient-related factors such as advanced age and female sex are also recognized contributors to increased susceptibility [1, 2].

Defining QTc prolongation is clinically important for stratifying risk. A QTc greater than 450 ms in men or greater than 470 ms in women is generally considered prolonged, while QTc ≥ 500ms are strongly associated with a heightened risk of TdP and sudden cardiac death [1, 5]. Such thresholds serve as practical cut-offs for monitoring and therapeutic decision-making in high-risk populations.

Several observational studies have documented that one-quarter to one-third of ICU patients develop QTc prolongation during their admission [3, 4]. Importantly, new-onset QTc prolongation in ICU patients has been correlated with adverse outcomes, including elevated rates of ventricular arrhythmias, cardiac arrest, and mortality [3, 4]. These findings underscore that QTc prolongation in critically ill patients is not a benign epiphenomenon, but rather a marker of poor prognosis. However, most available data originate from North American, European, or East Asian cohorts [3]. There is a notable paucity of data from South Asian ICUs, despite differences in patient demographics, comorbidities such as diabetes and infectious diseases, and prescribing practices—for example, greater use of certain antimicrobials and antimalarials.

In view of these gaps, we conducted a prospective multicenter study in two tertiary-care ICUs in Pakistan to determine the incidence of ICU-acquired QTc prolongation and to identify its risk factors and clinical consequences. Our aim was to generate updated real- world data that could inform context-specific risk stratification and management strategies in South Asia. We hypothesized that approximately 30% of our ICU patients would develop QTc prolongation, and that this condition would be associated with identifiable predictors such as sepsis, multiorgan dysfunction, and exposure to QTc-prolonging medications, in line with evidence reported in other populations [3, 5]. Recent local evidence further highlights the clinical relevance of QTc risk stratification in Pakistan. Humza et al. [21] conducted a pharmacist-led interventional study in a specialized cardiac ICU, applying the Framingham heart rate correction formula and the Tisdale risk scoring systems for identifying QTc prolongation and risk stratification. Their study demonstrated a measurable reduction in the incidence of QTc prolongation after implementation of a structured QTc-risk protocol.

However, their work was limited to a single cardiac center and focused on prevention to intervention rather than identification of independent clinical predictors.

Our study expands on these findings by examining a broader, mixed medical-surgical ICU population across two tertiary-care hospitals, aiming to determine the incidence, predictors, and clinical consequences of ICU-acquired QTc prolongation in routine practice settings [21].

The objective of this study was to determine the incidence of new-onset QTc interval prolongation in critically ill patients admitted to intensive care units and to identify the clinical and pharmacologic factors associated with its development.

## Methods

Study Design and Setting: This was a prospective, observational cohort study conducted in the medical-surgical ICUs of two tertiary care hospitals in Pakistan (one in Lahore, one in Karachi) over 12 months. The study was approved by each institution’s ethics review board, and informed consent was obtained from patients or surrogates according to local protocols. We adhered to international ethical guidelines for human research and STROBE reporting standards.

### Study endpoints

The primary endpoint of this study was to identify independent clinical and pharmacologic predictors of ICU-acquired QTc prolongation among critically ill patients.

The secondary endpoints included:

1. Determination of the incidence and timing of new-onset QTc prolongation during ICU stay.
2. Evaluation of the frequency of severe QTc prolongation (≥ 500 ms) and its association with arrhythmic events.
3. Comparison of ICU length of stay and in-hospital mortality between patients with and without QTc prolongation.

Patient Selection: We enrolled consecutive adult patients (age ≥ 18) admitted to the ICUs. Patients were excluded if they had a pre-existing prolonged QTc or conditions that would preclude accurate QTc measurement: namely, known congenital long QTc syndrome, baseline QTc > 450 ms in men or > 470 ms in women on the admission ECG, presence of a pacemaker or bundle-branch block, or if they were on chronic ventricular pacing (which obscures QTc duration). We also excluded patients who stayed in the ICU < 24 h or had incomplete ECG data. These criteria ensured we captured new-onset (acquired) QTc prolongation developing in ICU rather than persistent long QTc present before admission.

Enrolled patients underwent serial ECG assessments throughout their ICU stay. A standard 12-lead ECG was obtained at baseline on ICU admission, and then at predefined intervals (daily or every other day), as well as whenever there was a significant clinical change or the initiation of high-risk medication. Continuous cardiac telemetry with centralized monitoring was maintained for all patients.

The QTc was measured from the onset of the QRS complex to the end of the T wave on lead II or V5, whichever provided a clearer T-wave termination. QTcs were corrected for heart rate using Bazett’s formula (QTc = QTc/√RR) [1]. Recognizing that Bazett’s method tends to overestimate QTc at higher heart rates [2], measurements were preferentially taken during rate-stable periods or after heart rate control had been achieved. For consistency and reliability, all QTc values were reviewed independently by a cardiologist.

QTc prolongation was defined as > 450 ms in men and > 470 ms in women [3], consistent with widely accepted thresholds. Severe prolongation, defined as ≥ 500 ms, was also noted, given its strong association with TdP and sudden cardiac death [4].

### Data collection

We recorded comprehensive baseline demographic and clinical characteristics, including age, sex, comorbidities (with emphasis on cardiovascular diseases such as coronary artery disease and heart failure), and illness severity using the Acute Physiology and Chronic Health Evaluation II (APACHE-II) score. ICU course data encompassed the primary admission category (medical, surgical, or mixed), presence of sepsis or septic shock (as per Sepsis-3 definitions), and the occurrence of organ dysfunction. Particular attention was paid to acute kidney injury (AKI), classified according to KDIGO criteria, as well as the need for renal replacement therapy and mechanical ventilation.

Daily laboratory data included serum potassium, magnesium, and ionized calcium. Episodes of hypokalemia (< 3.5 mmol/L) and hypomagnesemia (< 1.6 mg/dL), both well-established precipitants of QTc prolongation, were carefully documented [5].

### Confounding variables and covariates

To ensure accurate identification of independent predictors, a set of a priori confounding variables was defined based on established pathophysiologic and pharmacologic mechanisms influencing QTc duration. These included:

- Electrolyte disturbances: hypokalemia (< 3.5 mmol/L), hypomagnesemia (< 1.6 mg/dL), and hypocalcemia (< 8.5 mg/dL).
- Medication exposures: use of known or possible QTc-prolonging drugs (as per AZCERT classification), use of loop diuretics (e.g., furosemide, bumetanide) due to their potential to cause electrolyte loss, and use of key ICU sedatives (haloperidol, dexmedetomidine, morphine, fentanyl).
- Organ dysfunction: acute kidney injury (AKI) according to KDIGO criteria, and hepatic dysfunction, defined by elevated transaminases (> 3× ULN) or total bilirubin > 2 mg/dL, given their effects on drug clearance and metabolism.
- Systemic conditions: presence of sepsis or septic shock (per Sepsis-3 definitions) and multiple organ dysfunction syndrome (MODS).
- Cardiac comorbidities: pre-existing coronary artery disease (CAD), heart failure, or structural heart disease documented before ICU admission.
- Demographics: age, sex, and illness severity (APACHE-II score) these variables were included in univariate analyses, and those with *p* < 0.10 were entered into the multivariable logistic regression model along with clinically relevant covariates identified a priori.

Medication exposure was meticulously tracked. All drugs administered with known or potential QTc-prolonging effects were classified using the CredibleMeds© database. Agents included antiarrhythmics (e.g., amiodarone), macrolide and fluoroquinolone antibiotics, antifungals (fluconazole), antiemetics, and antipsychotics (particularly haloperidol, commonly prescribed for ICU delirium). Sedatives such as dexmedetomidine were also recorded, since prior reports suggest it has minimal QTc effect [6]. For each patient, we quantified the cumulative number of QTc-prolonging drugs received, and flagged specific high-risk agents such as haloperidol, quetiapine, and methadone. For each patient, a **Tisdale Risk Score** was calculated to quantify the risk of drug-induced QTc prolongation. The score incorporates demographic factors (age ≥ 68 years, female sex), clinical variables (loop diuretic use, baseline QTc ≥ 450 ms), and medication-related factors (≥ 2 QTc-prolonging drugs, sepsis, hypokalemia). Patients were stratified as low (≤ 6), moderate (7–10), or high risk (≥ 11) based on total points [Tisdale et al., Circ Cardiovasc Qual Outcomes 2013;6:479–487].

### Medication exposure

All QTc-prolonging medications were classified according to the AZCERT (CredibleMeds^®^) database, which stratifies drugs into four risk categories:

- *Known Risk (KR):* Drugs with substantial evidence of causing TdP (e.g., amiodarone, haloperidol, methadone).
- *Possible Risk (PR):* Drugs that can prolong QTc but lack conclusive evidence for torsades (e.g., azithromycin, fluconazole).
- *Conditional Risk (CR):* Drugs that cause QTc prolongation only under specific conditions such as overdose or drug–drug interaction (e.g., ondansetron, quetiapine).
- *Drugs to Avoid in Congenital Long QTc (CA):* Those contraindicated in congenital LQTcS but not otherwise high-risk (e.g., sertraline).
- The classification for each medication administered during ICU stay was verified using the Credible Meds database (accessed January 2025), and cross-checked by a clinical pharmacist before analysis as shown in Table 1 in results section.

**Table 1:** QTc-prolonging drugs administered during ICU stay classified by AZCERT (CredibleMeds) categories

AZCERT risk category	Representative drugs used in study cohort	Frequency of use (*n*, %)
Known Risk (KR)	Haloperidol, Amiodarone, Methadone	210 (25.9%)
Possible Risk (PR)	Azithromycin, Fluconazole	165 (20.3%)
Conditional Risk (CR)	Ondansetron, Quetiapine	120 (14.8%)
Drugs to Avoid in Congenital LQTcS (CA)	Sertraline, Escitalopram	60 (7.4%)
No Known QTc Risk	Dexmedetomidine, Midazolam	257 (31.6%)

### Sample size and statistical analysis

Based on previous studies, we anticipated a QTc prolongation incidence of ~ 30% in ICU patients. A sample size of approximately 800 patients was calculated to provide > 80% power (α = 0.05) to detect a 10% difference in incidence between patients with and without predefined risk factors. Our final cohort of 812 patients was thus deemed statistically adequate. We prespecified an expected ICU-acquired QTc prolongation incidence of **~ 30%** based on prior ICU and hospitalized-patient data. Prospective ICU cohorts have reported QTc/LQTcS prevalences around **24–28% on admission** (e.g., **27.9%** in a 412-patient PLOS One study) and up to **24–61%** across ICU studies depending on definitions, timing, and monitoring intensity.^1^ In serial ICU assessments, incidence rose with length of stay (e.g., **6.5%** on day 1–**15.7%** by day 5).^2^ Moreover, in general hospitalized populations—the setting in which the Tisdale score was derived—**~30%** developed QTc > 500 ms or ΔQTc > 60 ms.³ Taken together, these data support using **30%** as a conservative, literature-grounded estimate for planning power in a mixed medical–surgical ICU.

Data analysis was performed using SPSS version 25.0 (IBM Corp., Armonk, NY). Continuous variables were expressed as mean ± standard deviation (SD) or median with interquartile range (IQR), depending on distribution, and compared using Student’s *t*-test or Mann–Whitney U test as appropriate. Categorical variables were reported as counts and percentages, with comparisons made using chi-square or Fisher’s exact tests.

A two-tailed *p*-value < 0.05 was considered statistically significant. Variables with *p* < 0.1 on univariate analysis, along with clinically relevant predictors (such as sex and age), were entered into a multivariable logistic regression model to identify independent predictors of new-onset QTc prolongation. Adjusted odds ratios (ORs) with 95% confidence intervals (CIs) were reported. Multicollinearity was assessed, and correlated variables (e.g., serum potassium and hypokalemia status) were not included simultaneously.

In a secondary analysis, clinical outcomes—including ICU length of stay and mortality—were compared between patients with and without QTc prolongation. Multivariable logistic regression was performed to evaluate whether QTc prolongation independently predicted ICU mortality after adjustment for illness severity and key covariates. Model calibration and goodness-of-fit were verified before final reporting.

## Results

Patient Characteristics: A total of 812 ICU patients were included after applying exclusion criteria. The cohort’s mean age was 56 ± 17 years, and 60% were male. Medical admissions comprised 40% of patients, surgical 35%, and 25% were mixed or other categories. Key baseline comorbidities included diabetes (32%), coronary artery disease (CAD, 20%), and congestive heart failure (10%). The median APACHE-II score at admission was 18 (IQR 12–25), reflecting a moderately high illness acuity. All patients had a normal QTc at ICU admission by study design (mean baseline QTc ~ 420 ± 25 ms).The median Tisdale score among patients who developed QTc prolongation was 10 (IQR 8–13) compared to 6 (IQR 4–9) in those who did not (*p* < 0.001). High Tisdale scores (≥ 11) were independently associated with a threefold increase in QTc prolongation (adjusted OR 3.2, 95% CI 2.0–5.0, *p* < 0.001).

**Fig. 1:**
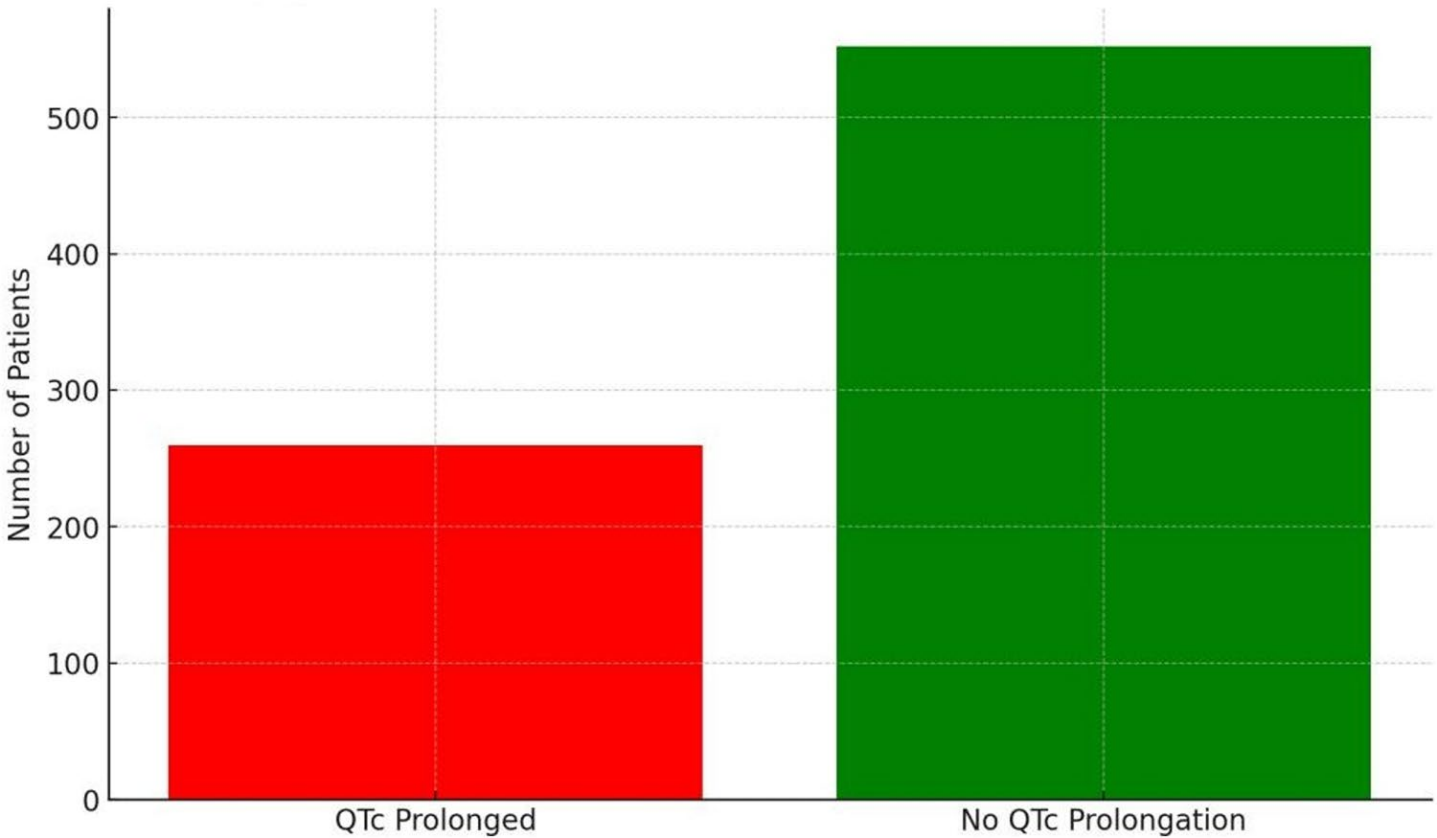
Incidence of QTc prolongation in ICU patient

Incidence of QTc Prolongation: During ICU stay, 260 of 812 patients developed QTc prolongation (incidence 32.0%). The majority of cases of QTc prolongation were first noted within the first week of ICU admission. Figure 1 illustrates the proportion of patients with new QTc prolongation observed over time. Figure 1: Incidence of new-onset QTc prolongation in the ICU cohort (32% of patients) is shown. This high incidence is in line with prior critical care studies [7, 8] underscoring that roughly one in three ICU patients experienced a prolonged QTc during their admission. Among the 260 patients who developed QTc prolongation, severe prolongation (QTc ≥ 500 ms) occurred in 48 patients (5.9% of the total cohort; 18.5% of those with QTc prolongation). Most of these cases were transient and associated with concurrent hypokalemia or hypomagnesemia. Two patients with QTc ≥ 500 ms experienced TdP, both resolving after electrolyte correction and withdrawal of QTc-active drugs.

Patients who developed QTc prolongation were compared with those who maintained normal QTc. Table 2 summarizes the key differences between these groups:

**Table 2:** The key difference between the two groups

Characteristic	QTc Prolongation (*n* = 260)	No QTc Prolongation (*n* = 552)	*p*-value
Sepsis (during ICU)	55%	30%	< 0.001
Septic shock	30%	12%	< 0.001
Acute Kidney Injury	45%	20%	< 0.001
Hypokalemia (K < 3.5 mmol/L)	35%	10%	< 0.001
Hypomagnesemia	30%	15%	< 0.001
IV Haloperidol use	22%	5%	< 0.001
Azithromycin use	18%	4%	< 0.001
Amiodarone use	12%	2%	< 0.001
Methadone use	5%	0%	< 0.001
ICU Length of Stay (days, median)	10 (IQR 7–15)	5 (IQR 3–8)	< 0.001
In-hospital Mortality	45%	18%	< 0.001

Table 2: Key differences in clinical factors and outcomes between patients with and without ICU-acquired QTc prolongation. Patients with prolonged QTc had significantly higher rates of sepsis and septic shock, organ failures (renal dysfunction), electrolyte derangements, and exposure to QTc-prolonging drugs compared to those who maintained normal QTc. They also had longer ICU stays and a greater mortality rate.

### Risk factor profile

As summarized in Table 2, patients who developed QTc prolongation demonstrated a markedly different risk profile compared with those who did not. Sepsis was a dominant factor: 55% of patients in the prolonged-QTc group had sepsis during their ICU course compared with 30% in the normal-QTc group (*p* < 0.001). More strikingly, 30% of the QTc- prolonged group experienced septic shock requiring vasopressor support versus 12% in the non-prolonged group, underscoring the interplay between circulatory failure, catecholamine surges, and repolarization abnormalities.

Acute kidney injury (AKI) was also significantly more common among patients with prolonged QTc (45% vs. 20%, *p* < 0.001). This aligns with the pathophysiological expectation that impaired renal clearance of drugs and concurrent electrolyte imbalances increase proarrhythmic susceptibility [1]. Electrolyte abnormalities were frequent triggers: 35% of the QTc-prolonged group developed significant hypokalemia compared with 10% in the normal- QTc group (*p* < 0.001), while hypomagnesemia occurred in 30% versus 15% (*p* < 0.001). These derangements often temporally coincided with QTc prolongation episodes, reinforcing their mechanistic role.

Traditional demographic risk factors were less influential. The proportion of females was similar in both groups (42% vs. 38%, *p* = 0.25), and mean age did not differ significantly (57 ± 17 vs. 55 ± 16 years, *p* = 0.10). Thus, in this critically ill population, acute clinical conditions outweighed intrinsic demographic predispositions.

### Medication exposure

Polypharmacy with QTc-liable drugs emerged as a major differentiator. In the prolonged- QTc group, 80% of patients received at least one QTc-prolonging drug compared with 59% in the non-prolonged group. The average number of QTc-risk drugs per patient was significantly higher in those with prolongation (1.8 vs. 0.9, *p* < 0.001).

Specific high-risk medications had clear associations. Intravenous haloperidol was administered to 22% of prolonged-QTc patients but only 5% of the normal-QTc group (*p* < 0.001). Quetiapine and other sedative antipsychotics were less commonly used overall (~ 5% of patients) but appeared disproportionately in the prolonged-QTc group. By contrast, dexmedetomidine was used in ~ 25% of patients across both groups, with no association with QTc prolongation, supporting prior evidence that its electrophysiologic profile is relatively neutral [2, 3].

Among antimicrobials, azithromycin was given to 18% of QTc-prolonged patients compared to 4% of those without prolongation (*p* < 0.001). Amiodarone, used for atrial fibrillation or refractory arrhythmias, was more common in the prolonged group (12% vs. 2%, *p* < 0.001). Methadone, although rarely used, was administered exclusively in the QTc-prolonged group (5%, *p* < 0.001). Other QTc-active drugs, such as fluconazole and levofloxacin, were infrequently used, but when present, were more often seen in patients who developed QTc prolongation.

### Multivariable analysis

Independent predictors of ICU-acquired QTc prolongation are shown in Table 3. The logistic regression model had good discrimination (C-statistic 0.78). Key predictors included:

- *Septic shock:* Adjusted OR 2.84 (95% CI 1.93–4.18, *p* < 0.0001). Septic shock nearly tripled the odds of QTc prolongation, consistent with catecholamine exposure, vasopressor therapy, and systemic inflammation.
- *Coronary artery disease (CAD):* OR 2.47 (95% CI 1.57–3.88, *p* < 0.001). Underlying CAD likely predisposes to repolarization heterogeneity and arrhythmogenic vulnerability [4].
- *Acute kidney injury (AKI):* OR 2.26 (95% CI 1.48–3.42, *p* < 0.001). Renal dysfunction contributes via impaired clearance of drugs and electrolyte disturbances [5].
- *QTc-prolonging drugs:* OR 1.39 per additional drug (95% CI 1.21–1.58, *p* < 0.0001). Each incremental QTc-risk medication conferred ~ 39% higher odds of prolongation, highlighting polypharmacy risk [6, 7].
- *Haloperidol use:* OR 3.18 (95% CI 1.88–5.37, *p* < 0.0001). IV haloperidol independently increased QTc prolongation risk more than three-fold, consistent with its known blockade of cardiac IKr channels [2].

Neither sex nor age retained significance in the adjusted model, underscoring the dominant role of acute illness and pharmacologic exposures in determining risk.

**Table 3:** Multivariable logistic regression analysis for independent predictors of ICU-acquired QTc prolongation (*n* = 812)

Variable	Adjusted odds ratio (OR)	95% Confidence interval (CI)	*p*-value
Septic shock	2.84	1.93–4.18	<0.0001
Coronary artery disease (CAD)	2.47	1.57–3.88	< 0.001
Acute kidney injury (AKI)	2.26	1.48–3.42	< 0.001
QTc-prolonging drugs (per additional agent)	1.39	1.21–1.58	< 0.0001
IV Haloperidol use	3.18	1.88–5.37	< 0.0001
High Tisdale risk score (≥ 11)	3.20	2.00–5.00	< 0.001
Female sex	1.12	0.83–1.62	0.25
Age > 60 years	1.18	0.82–1.71	0.31

### Clinical outcomes

QTc prolongation was strongly associated with adverse outcomes. Median ICU length of stay was twice as long in the prolonged-QTc group (10 vs. 5 days, *p* < 0.001). In-hospital mortality was significantly higher among patients with QTc prolongation (45% vs. 18%, *p* < 0.001).

After adjustment for APACHE-II score and other covariates, QTc prolongation remained an independent predictor of mortality (adjusted OR ~ 1.8, *p* = 0.048).

Arrhythmic complications were also more frequent. Non-sustained ventricular tachycardia occurred in 11% of QTc-prolonged patients versus 3% of others (*p* = 0.002). Polymorphic VT/TdP occurred in 0.8% of prolonged-QTc patients, both cases in the setting of QTc ≥ 500 ms with concurrent hypokalemia. Atrial fibrillation was also more frequent in the prolonged group (19% vs. 10%, *p* = 0.001). These findings align with prior sepsis-related studies linking QTc prolongation to increased arrhythmia burden [8, 9].

**Fig. 2:**
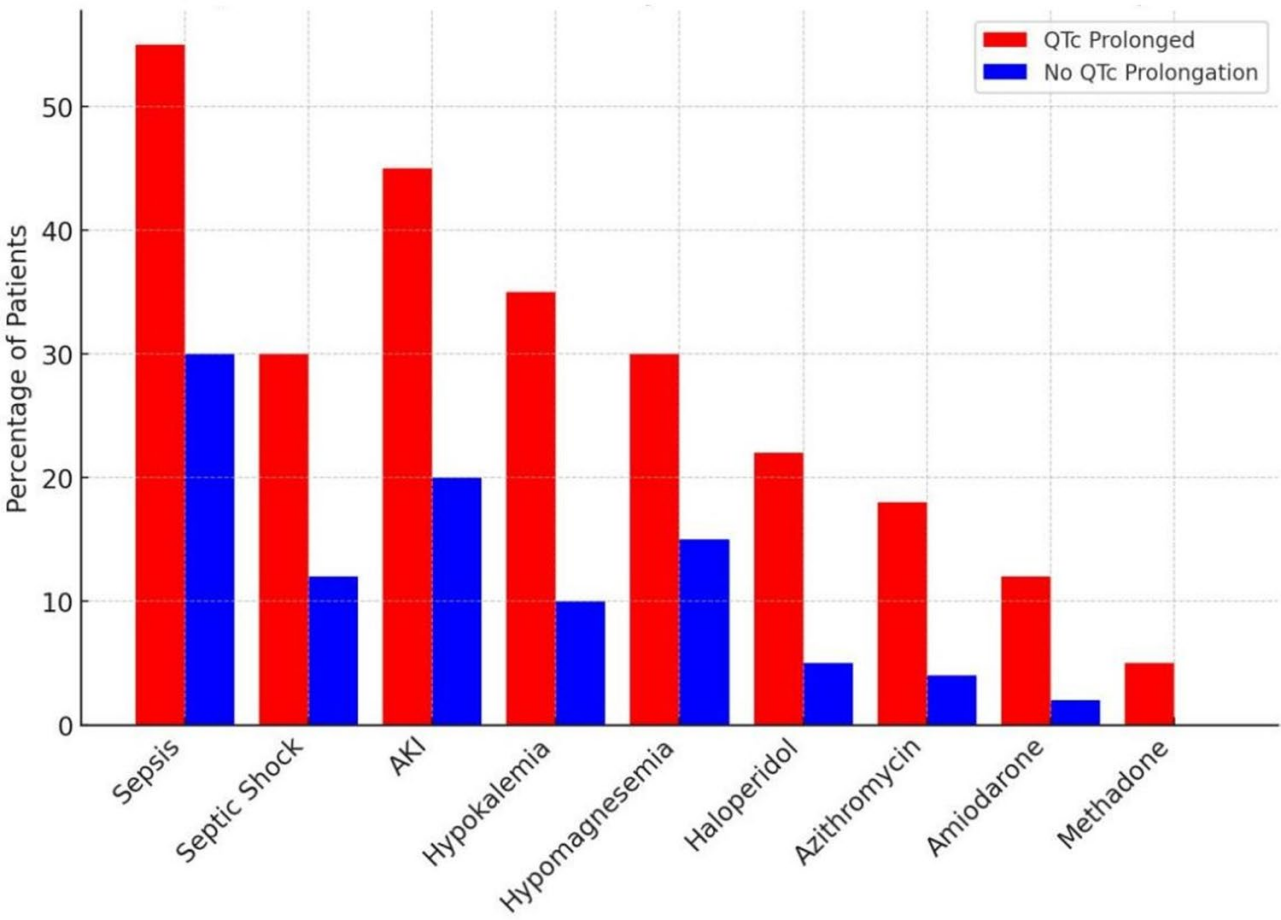
Distribution of key risk factors between groups

**Fig. 3:**
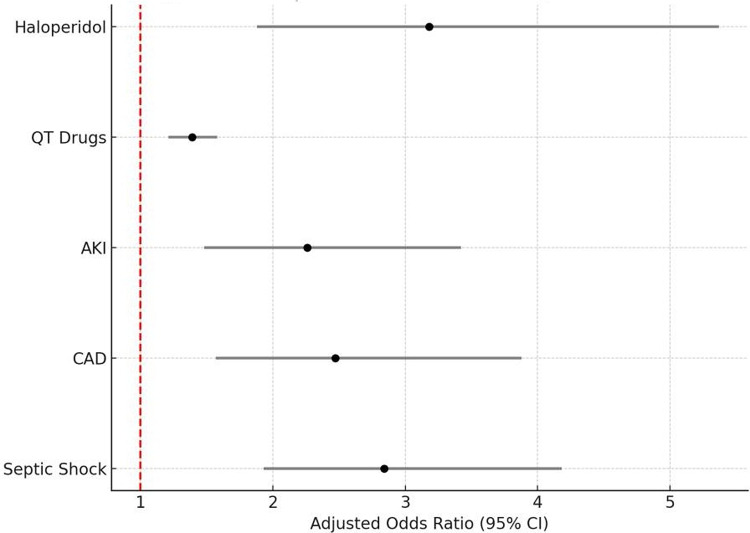
Independent predictors of QTc prolongation

Figures 2 and 3 visually demonstrate these associations: Fig. 2 highlights the distribution of key risk factors between groups, while Fig. 3 shows adjusted odds ratios for independent predictors, with septic shock, CAD, AKI, QTc-active drug burden, and haloperidol standing out as the strongest contributors.

### Tisdale risk score

The Tisdale Risk Score was calculated for all patients to estimate the risk of drug-induced QT interval prolongation. The mean score was 9.2 ± 2.1, with scores ranging from 4 to 15.

Based on risk categories, 22 (29%) patients had low risk (score ≤ 6), 39 (52%) had moderate risk (score 7–10), and 14 (19%) were classified as high risk (score ≥ 11). No statistically significant correlation was found between Tisdale score and QT prolongation events in this sample (*p* = 0.11) (Table 4).

**Table 4:** Tisdale risk score categories

Tisdale risk category	Score range	No. of patients (*n* = 75)	Percentage (%)
Low risk	≤ 6	22	29%
Moderate risk	7–10	39	52%
High risk	≥ 11	14	19%

## Discussion

### Overview and context

In this multicenter prospective study of ICU patients in Pakistan, we demonstrated that new-onset QTc prolongation occurred in approximately one-third of critically ill patients. This frequency is slightly higher but broadly consistent with prior reports from other regions, where estimates have ranged from 20 to 30% [1, 2]. The novelty of our findings lies in their contribution to the limited data from South Asian ICUs, an important gap given regional differences in demographics, prescribing patterns, and comorbidity profiles. Crucially, our results show that QTc prolongation in ICU is not merely an electrocardiographic curiosity—it carries significant prognostic implications, including a longer ICU length of stay and markedly higher in-hospital mortality. These findings align with work by Liang et al. [15], who observed that QTc prolongation in septic patients predicted higher 30-day mortality [3, 4].Use of the Tisdale score allowed objective risk quantification, aligning with prior ICU studies demonstrating its predictive utility for QTc prolongation [Tisdale 2013; Sadlonova 2025]. Incorporating such scoring systems may enhance early detection and prevention.

### Sepsis and septic shock

Among all risk factors, septic shock emerged as one of the strongest independent predictors of QTc prolongation. Sepsis is characterized by systemic inflammation, cytokine storms, oxidative stress, and autonomic dysregulation, all of which can impair myocardial repolarization [5, 6]. In particular, cytokine-mediated modulation of potassium currents and acidosis-induced alterations in calcium handling are plausible mechanisms for QTc prolongation during septic states. Liu et al. [1] highlighted that respiratory, cardiac, and renal dysfunction often co-exist with sepsis, further compounding the risk of repolarization abnormalities [7]. Importantly, they reported that sepsis-related QTc prolongation could mimic congenital long QTc syndromes [8]. In our cohort, over half of patients with QTc prolongation were septic, reinforcing that clinicians should be highly vigilant for QTc abnormalities in this subgroup. Moreover, ICU treatments commonly used in septic shock— such as high-dose catecholamines and QTc-active antibiotics—further amplify this risk.

### Acute kidney injury (AKI)

Renal dysfunction was another powerful independent predictor. AKI contributes to QTc prolongation by impairing clearance of renally excreted drugs, resulting in higher plasma drug levels, while also causing electrolyte derangements (e.g., hypo- and hyperkalemia, hypomagnesemia). These changes disrupt myocardial repolarization and predispose to TdP [9]. While chronic kidney disease is well known to confer arrhythmic risk, our study adds to evidence that acute renal failure in ICU is a potent, dynamic risk factor for QTc prolongation. This finding suggests that early nephrology input and aggressive electrolyte optimization may be important preventive measures.

### Medication-associated risk

The association between haloperidol use and QTc prolongation was especially striking. We found an adjusted OR of ~ 3.2 for IV haloperidol, affirming its potent pro-arrhythmic potential [10]. Wang et al. [8] reported similar risks for haloperidol and quetiapine, especially when combined with electrolyte abnormalities or other QTc-risk drugs [11, 12]. In our ICU cohort, haloperidol was often prescribed for delirium in critically ill patients already carrying multiple QTc risk factors, thereby compounding risk. Although interventional trials such as MIND-USA reported no significant QTc effect when haloperidol was carefully monitored in lower-risk populations [13, 14], our results highlight that in real-world ICU practice—where patients are more unstable and monitoring may be less intensive—haloperidol can significantly increase QTc. Current guidelines recommend close monitoring and dose minimization, and our results support preferential use of alternatives such as dexmedetomidine in high-risk patients [15]. The distribution of QTc-prolonging drugs in our cohort was dominated by **AZCERT “known-risk” agents**, particularly haloperidol and amiodarone. This pattern emphasizes the need for integrating AZCERT-based risk classification into ICU prescribing workflows to identify and mitigate avoidable QTc exposure.

### Polypharmacy and drug interactions

Polypharmacy was another key determinant: each additional QTc-risk medication raised the odds of QTc prolongation by ~ 40%. This cumulative effect is highly clinically relevant, as most ICU patients are exposed to complex drug regimens. Prior retrospective studies have similarly shown that polypharmacy drives QTc risk, with more than half of ICU patients receiving at least one QTc-prolonging drug [16, 17]. Clinical decision support systems, pharmacist-led reviews, and QTc risk scoring (e.g., Tisdale score) have been shown to mitigate risk by flagging dangerous drug combinations [18]. In our ICUs, we implemented an internal protocol mid-study to discontinue all non-essential QTc-active drugs once QTc exceeded 500 ms, substituting safer alternatives whenever feasible.

### Dexmedetomidine as a safer alternative

An important observation was that dexmedetomidine was not associated with QTc prolongation. Unlike haloperidol, it has no significant IKr channel blockade and may even have protective electrophysiologic effects by reducing catecholamine surges [19, 20]. Haspolat et al. [5] directly compared haloperidol and dexmedetomidine in delirious ICU patients, showing that haloperidol increased QTc by ~ 18 ms while dexmedetomidine caused no prolongation [21]. Our findings corroborate this and support the growing ICU practice of favoring dexmedetomidine over haloperidol, especially in high-risk patients.

### Electrolyte management

Electrolyte abnormalities, particularly hypokalemia and hypomagnesemia, were strongly associated with QTc prolongation. More than one-third of QTc-prolonged patients developed hypokalemia, often in temporal association with QTc changes. These findings emphasize that rigorous electrolyte repletion—targeting potassium ≥ 4.0 mmol/L and magnesium ≥ 2.0 mg/dL—is an essential preventive strategy [21, 22]. Given that magnesium sulfate is first-line therapy for TdP, routine monitoring and proactive supplementation are both evidence-based and low-cost interventions.

### Clinical outcomes

QTc prolongation was not merely an epiphenomenon of critical illness but an independent predictor of worse outcomes. Patients with QTc prolongation had double the ICU length of stay and over twice the in-hospital mortality compared to those without. Even after adjusting for APACHE-II scores, QTc prolongation retained prognostic significance. Arrhythmias—including nonsustained VT, atrial fibrillation, and rare cases of TdP—were significantly more frequent in the QTc-prolonged group [23, 24]. These findings underscore the need to treat QTc prolongation as a clinically meaningful warning sign rather than a benign incidental finding.

### Comparative context

Our incidence of 32% is higher than some reports, such as Farzanegan et al. [2] in Iran, who found 15.7% prevalence by day 5, and Fernandes et al. [2] in Brazil, who reported 28% [21]. Differences likely stem from inclusion criteria, drug use patterns (e.g., high haloperidol and fluoroquinolone/macrolide use in our cohort), and monitoring practices. We also observed a strong association between CAD and QTc prolongation, consistent with prior data linking structural heart disease to increased arrhythmic risk.

The incidence of QTc prolongation in our cohort (32%) was notably higher than that reported by Humza et al. [21], who observed a 14.6% rate in a pharmacist-driven cardiac ICU population. Several factors likely explain this discrepancy [21].

First, our study enrolled a broader spectrum of critically ill patients, including both medical and surgical cases, whereas Humza et al. focused on a more homogeneous cardiac ICU population where baseline QTc monitoring and cardiology oversight were routine.

Second, their pharmacist-led protocol proactively applied Framingham and Tisdale risk scores to modify or discontinue high-risk medications early, effectively reducing QTc events. In contrast, our study was purely observational, capturing real-world practice without such structured intervention, allowing natural incidence to emerge.

Third, differences in medication patterns may have contributed—our ICUs used haloperidol, macrolides, and amiodarone more frequently than cardiac ICUs, where antiarrhythmic regimens are tightly regulated.

Lastly, inter-hospital variability in electrolyte monitoring frequency and replacement protocols may also have amplified the observed incidence. Together, these contextual differences suggest that our higher rate reflects less standardized QTc surveillance and polypharmacy typical of mixed ICUs, rather than a true population-level difference in susceptibility.

Our observed incidence (32%) falls within published ICU ranges and near the ≈ 30% rate seen in hospitalized cohorts used to derive QTc-risk tools, consistent with variability related to population mix, timing, and monitoring intensity.^1–2^.

In another Pakistani observational study, Khalid et al. (2023) evaluated ECG abnormalities among hospitalized COVID-19 patients at a tertiary-care center and noted that QTc prolongation occurred in approximately one-fifth of patients, mainly associated with azithromycin and hydroxychloroquine use. Although their focus was infection-related QTc risk in medical wards rather than ICU settings, their findings parallel ours in demonstrating the contribution of antibiotic exposure and electrolyte derangements to QTc changes [21].

Together, these Pakistani studies underscore that QTc prolongation is a clinically relevant and under-recognized problem in local hospitals. Our study builds on this groundwork by providing the first prospective, multicenter ICU-based dataset quantifying incidence, risk factors, and outcomes, thereby contextualizing global evidence within South Asia.

### Practical clinical implications

Our findings suggest a multi-pronged approach for ICU teams:

1. Routine QTc monitoring in high-risk patients (sepsis, shock, polypharmacy, organ failure).
2. Aggressive electrolyte management with potassium and magnesium optimization.
3. Judicious drug prescribing, avoiding haloperidol and polypharmacy where possible, and favoring safer alternatives such as dexmedetomidine.
4. Pharmacist-driven reviews of drug regimens for QTc interactions.
5. Risk stratification tools like the Tisdale score to guide monitoring intensity [18].
6. Multidisciplinary “QTc alert” systems when QTc exceeds 500 ms, ensuring timely adjustments.

Our findings complement the recent work of Humza et al. [21], who implemented a pharmacist-led QTc-risk assessment protocol using Framingham and Tisdale scores in a cardiac ICU. While their intervention reduced the rate of QTc prolongation through proactive medication management, our multicenter observational data show that even in the absence of structured scoring, similar risk determinants—such as sepsis, acute kidney injury, and polypharmacy—remain predominant. Together, these studies underscore the importance of combining continuous ECG surveillance with systematic QTc-risk assessment to prevent arrhythmic complications in critically ill patients [21].

## Limitations

Our study was observational, limiting causal inference. QTc was measured using Bazett’s formula, which may overestimate QTc at high heart rates. While we excluded baseline bundle branch block or pacing, this may limit generalizability. Finally, our study was limited to two ICUs in Pakistan, and results may not be universally generalizable.

## Conclusion

Nearly one in three critically ill patients in our Pakistani multicenter cohort developed QTc prolongation, strongly linked to septic shock, AKI, and use of multiple QT-prolonging drugs—especially haloperidol. Dexmedetomidine showed no association with QTc prolongation, suggesting a safer alternative. Prolonged QTc was associated with higher arrhythmia and mortality risk, confirming its prognostic value. Rigorous electrolyte correction, judicious drug selection, and routine ECG monitoring are essential, and rising QTc should be treated as an early ICU warning sign, similar to lactate or creatinine.

## Data Availability

No datasets were generated or analysed during the current study.
